# Plasma Rich in Growth Factors in Macular Hole Surgery

**DOI:** 10.3390/clinpract12010007

**Published:** 2022-01-10

**Authors:** Ronald M. Sánchez-Ávila, Carlos A. Robayo-Esper, Eva Villota-Deleu, Álvaro Fernández-Vega Sanz, Álvaro Fernández-Vega González, Borja de la Sen-Corcuera, Eduardo Anitua, Jesús Merayo-Lloves

**Affiliations:** 1 Ophthalmology Research Foundation, Fernández-Vega University Institute, University of Oviedo, 33071 Oviedo, Spain; carlosrobayo@hotmail.com (C.A.R.-E.); milideleu@yahoo.com (E.V.-D.); afdzvega@gmail.com (Á.F.-V.S.); afdezvega@gmail.com (Á.F.-V.G.); merayo@fio.as (J.M.-L.); 2 Regenerative Medicine Laboratory, Biotechnology Institute (BTI), 01007 Vitoria, Spain; bdelasen@bti-health.com (B.d.l.S.-C.); eduardoanitua@eduardoanitua.com (E.A.); 3 Regenerative Medicine Laboratory, University Institute for Regenerative Medicine and Oral Implantology (UIRMI), 01007 Vitoria, Spain

**Keywords:** macular hole, PRGF, retinal regeneration, optical coherence tomography, macular hole surgery

## Abstract

The aim of this study was to evaluate the use of PRGF (plasma rich in growth factors) as an adjuvant to PPV (pars plana vitrectomy) in recurrent, persistent, or poor prognosis MH (macular hole). Patients with MH were treated with PPV plus adjuvant therapy (PRGF membrane (mPRGF) and injectable liquid PRGF (iPRGF)). The anatomical closure of MH and postoperative BCVA (best-corrected visual acuity) were evaluated. Eight eyes (eight patients) were evaluated: myopic MH (MMH, *n* = 4), idiopathic MH (IMH, *n* = 2), iatrogenic *n* = 1, traumatic *n* = 1. The mean age was 53.1 ± 19.3 years. Hence, 66.7% (*n* = 4) of patients previously had internal limiting membrane peeling. Five patients (62.5%) received mPRGF and iPRGF, and three patients (37.5%) received iPRGF. Gas tamponade (C3F8) was placed in seven cases and one case of silicone oil. Anatomic closure of MH was achieved in seven eyes (87.5%) and BCVA improved in six cases. In the MMH group, visual acuity improved in two lines of vision. Follow-up time was 27.2 ± 9.0 months. No adverse events or MH recurrences were recorded during follow-up. The use of PRGF as an adjuvant therapy to PPV can be useful to improve anatomical closure and visual acuity in MH surgery.

## 1. Introduction

The macular hole (MH) is an anatomical defect in the retina located in the fovea center, which causes a significant decrease in vision [1]. The estimated prevalence in the general population is approximately 3.3 per 1000 inhabitants [2], mainly affecting people over 65 years of age and with two thirds corresponding to women [3]. The MH can be divided into two categories, namely primary or idiopathic (IMH) and secondary [3]. The IMH is caused by vitreous traction in the center of the fovea in the anteroposterior and tangential direction [1]. Classically, it has been classified in Gass stages (0–4). However, this classification is currently expanded according to the size of MH measured by OCT (optical coherence tomography) [4]. The secondary MH can be caused by various conditions, such as trauma, myopia, macular schisis, diabetic macular oedema, age-related macular degeneration, central venous occlusion of the retina, or type 2 macular telangiectasias [3].

The standard gold treatment for MH is pars plana vitrectomy (PPV), with the release of the posterior vitreous, associated or not with peeling of the internal limiting membrane (ILM), together with the use of a gas tamponade [5]. Another method used in surgery is the autologous ILM plug, with favorable clinical results in large MHs [6], persistent full-thickness MHs [7], and refractory MHs [8]. Recurrent IMH rates vary from 8% to 44% according to some published studies, possibly related to MH size (>400 µm), the presence of subretinal fluid, and retinal detachment [3,9]. In the myopic macular hole (MMH), the anatomical success achieved after PPV can be between 62.5% and 87.5%, according to some published series, frequently requiring a second intervention to achieve MH closure [10,11]. 

To increase the success rate in MH closure, several adjuvant therapies to PPV have been described: transforming growth factor-beta 2 as a chorioretinal adhesive [12], autologous serum, thrombin, and autologous platelet concentrates [13,14,15]. Plasma rich in growth factors (PRGF) is an autologous blood-derived product, with calcium-based plasma activation and four ophthalmological formulations (injectable liquid, eye drops, clot, and membrane) [16,17]. It has a closed production system, with predictability in its manufacture and safety in its use [18]. PRGF has demonstrated anti-inflammatory, anti-fibrotic, and regenerative functions in ocular surface and cornea diseases (dry eye, persistent epithelial defects, neurotrophic keratitis, among others) [19,20,21,22]. The use of the PRGF membrane in the closure of MH in high myopia has also achieved good anatomical and visual results [23]. 

The use of blood derivatives as an adjunct to IMH surgery has been widely investigated even in clinical trials [24]. However, PRGF is still under evaluation in the treatment of large, recurrent, persistent, or poor prognosis MHs in clinical follow-ups longer than one year. This study aims to describe the anatomical and visual results of the use of PRGF as an adjunct to PPV in patients with MH (large, recurrent, persistent or of poor prognosis). Moreover, we aim to evaluate the restoration potential of the outer retinal layers by OCT images. 

## 2. Materials and Methods

This retrospective, single-center, interventional, and case-series study was conducted at the Fernández-Vega University Institute (Oviedo, Spain). The Institutional Review Board approved this study. Patients provided informed consent for the surgical procedure and the use of PRGF. The principles of the Declaration of Helsinki were followed. 

### 2.1. Patients

Patients were included between 2011 and 2018 who were required to have a diagnosis of persistent MH (no primary closure after PPV), recurrent MH (reopening of MH after 3 months of PPV), or poor prognosis of closure due to its size (MH large > 400 µm of diameter, measured at the narrowest point of the hole). MHs of diverse etiology were selected: myopic, iatrogenic, idiopathic, and traumatic. A complete pre-surgical evaluation was performed, including demographic data, ophthalmological history, slit-lamp examination of the ocular surface, cornea, and fundus in pharmacological dilatation. Data were recorded before and after surgical treatment with PRGF: best-corrected visual acuity (BCVA) was measured in decimals (transformed to LogMAR: logarithm of the minimal angle of resolution); intraocular pressure (IOP) was measured with Goldmann applanation tonometry; analysis was performed by spectral-domain OCT (Cirrus 5000, Carl Zeiss AG, Oberkochen, Germany), making three measurements of MH (minimum diameter, base diameter, height), state of the lens, and MH closure. Gained visual acuity lines were also evaluated.

The MH diagnosis time (months) and the time with the MH opening until surgery with PRGF was performed (months) were also recorded. Additionally, safety data associated with the treatments administered to the patients were recorded. The minimum diameter is the smallest distance between the edges of the MH, and allows its size to be classified as small (<250 μm), medium (250–400 μm), and large (>400 μm) [25]. The base diameter corresponds to the distance between the two edges of the MH at the level of the retinal pigment epithelium (RPE), and the height is the average of the measurements between the RPE and the ILM at the edges of the hole. 

### 2.2. Preparation of PRGF

To prepare the PRGF membrane (mPRGF), 81 mL of peripheral blood was drawn in 9 mL tubes with 3.8% sodium citrate as anticoagulant. Subsequently, the blood was centrifuged at 580× *g* at room temperature for 8 min, using the Endoret closed system (PRGF, Ophthalmology kit, BTI Biotechnology Institute, S.L., Miñano, Álava, Spain). The manufacturing protocol for the PRGF injectable liquid and membrane manufacturing protocol described by Anitua and Col was followed [21,26]. The plasma column formed was divided into two fractions: Fraction 2 (F2), defined as the 2 mL above the leukocyte layer; and Fraction (F1), as the remaining plasma above F2. The F2 fraction was used to prepare the mPRGF, requiring 5 mL of F2 previously activated with 10% calcium chloride Endoret Activator (BTI Biotechnology Institute, S.L., Miñano, Álava, Spain) and incubated in 35 mm vials for 30 min at 37 °C with the Plasmaterm H Heater (BTI Biotechnology Institute). The clot formed was transferred to a shaper and pressed for the 30 s in order to obtain a 100 µm thick membrane. The mPRGF was placed on nitrocellulose disks to be used in the surgery room. The F1 fraction was also activated with Endoret Activator immediately before being used as an injectable PRGF liquid (iPRGF) during surgery. 

### 2.3. The Surgical Technique Using PRGF in the Macular Hole 

The 10% povidone-iodine was used for cleaning the ocular surface (10 min before anesthesia application). Retrobulbar anesthesia was performed (mepivacaine hydrochloride, 2%; B. Braun Melsungen AG; Arnhem, The Netherlands) in all patients, using a maximum of 4 mL of anesthetic. A complete PPV was performed using 23-gauge valved trocars (Constellation Vision System; Alcon Surgical, Fort Worth, TX, USA); when necessary, endoillumination with a chandelier cannula was used to facilitate bimanual movements. If ILM was present, it was stained with brilliant blue G (Dutch Ophthalmic Research Center BV, Zuidland, The Netherlands). In cases with peeling of internal limiting membrane (P-ILM), an extension of the ILM rhexis and transposition were performed according to the surgeon’s criteria. The mPRGF plug was trimmed and rolled for later insertion across to 23-gauge trocars. mPRGF could be manipulated inside the vitreous cavity and placed under perfluorocarbon liquid (PFCL, Carl Zeiss Meditec AG, Jena, Germany) inside the MH. In some cases, iPRGF was added (3 drops, leaving it to act for 5 min to allow a better adhesion). The decision to place the mPRGF or iPRGF was based on the clinical judgment of the surgeon. PFCL-fluid-air exchange was performed at the end of the surgery, leaving gas tamponade (C3F8) or standard silicone oil (Oxane 1300; Bausch + Lomb, Incorporated, Rochester, NY, USA) according to the surgeon’s criteria and subsequently placing the patient for 1 h in the supine position immediately after surgery. The patient had to be maintained in the prone position for the next three days and gradually moved towards the supine position over the following two weeks. There were no intraoperative complications with the surgical technique used. Patients received topical antibiotics and anti-inflammatory treatment during the month after surgery, with a gradual decrease according to protocol. Patients had a minimum follow-up of 6 months to be included in the study. 

### 2.4. Statistical Analysis

Descriptive statistics were performed using absolute and relative frequencies for qualitative variables and the mean, and standard deviation was used for quantitative variables. The level of statistical significance was at *p* < 0.05. The statistical software used was SPSS v20.0 for Windows (SPSS Inc., Chicago, IL, USA), and Excel 14.0 software was also used (Microsoft Office 2011, Microsoft Corp, Albuquerque, New Mexico).

## 3. Results

Eight patients (eight eyes) with MH of different etiology were included. Four were MMH (50.0%), two eyes were IMH (25.0%), one was of iatrogenic origin (12.5%), and one was traumatic (12.5%). The overall mean age was 53.1 ± 19.3 (15.0–71.0) years, the mean age in men was 46.2 ± 22.0 (15.0–71.0) years, and for women was 64.7 ± 3.1 (62.0–68.0) years. Fifty per cent were right eyes, and 50% left eyes. Men correspond to 62.5% (*n* = 5) and women to 37.5% (*n* = 3). The time with the initial diagnosis of MH was as follows: overall mean of 19.9 months; for MMH, it was 30.8 months; in IMH 13.5 months, in iatrogenic three months, and for the trauma, it was six months. Two eyes (25%) previously had two surgeries with failure to close the MH, four eyes (50%) had one surgery without achieving MH closure, and two eyes (25%) had not had previous surgeries (IMH, traumatic). Of those who had previous surgeries, 66.7% (*n* = 4) had P-ILM. In all previously operated patients, the gas tamponade was C3F8. The mean time between reopening of the MH until the surgery using the PRGF is 16.6 ± 20.7 (1.6–63.0) months, where the longest time (63.0 months) corresponds to a case of MMH and the shortest time (1.6 months) was iatrogenic (Table 1). All eyes with MMH had previous unsuccessful surgeries with MH recurrence. The average time with a diagnosis of MH for this group was 30.8 ± 29.2 (3.0–72.0) months, and the mean time between MH recurrence until surgery with PRGF was 24.8 ± 26.6 (2.8–63.0) months. 

For patient 1, when separating the hyaloid during the first PPV, strong adherence to the fovea was found, which when separated caused an iatrogenic MH. Patient 2 has a complete vision loss in the contralateral eye due to multiple atrophic myopic scars. Before surgery with PRGF, seventy-five per cent of eyes were pseudophakic (*n* = 6). The initial BCVA (logMAR) for the MMH group was 0.837 ± 0.383 (0.523–1.000) and the initial IOP was 13.3 ± 1.3 (12–15). In the OCT measurements, it was found that the overall mean of the base diameter was 1181.6 ± 345.7 (673.0–1577.0) µm, for the minimum diameter it was 516.9 ± 154.7 (313.0–806.0) µm, and for the height it was 462.8 ± 58.1 (380.0–519.0) µm. In the MMH group, these measurements were: base diameter 1049.5 ± 416.8 (673.0–1470.0) µm, minimum diameter 531.5 ± 229.6 (313.0–806.0) µm, and height 454.0 ± 59.3 (389.0–511.0) µm. As for the surgeries performed on the four eyes that previously had P-ILM, two underwent only ILM rhexis and the other two rhexis plus ILM transposition into the MH. Hence, 37.5% (*n* = 3) received iPRGF and 62.5% (*n* = 5) received m/iPRGF (Table 2). Patients 3 and 5 needed to peel the epiretinal membrane (ERM) with which they had been associated. Patient 3 had the silicone oil removed at 3.7 months, leaving the final BCVA at 0.1 (decimal), having previously started with BCVA of 0.05 (decimal).

**Figure 1:**
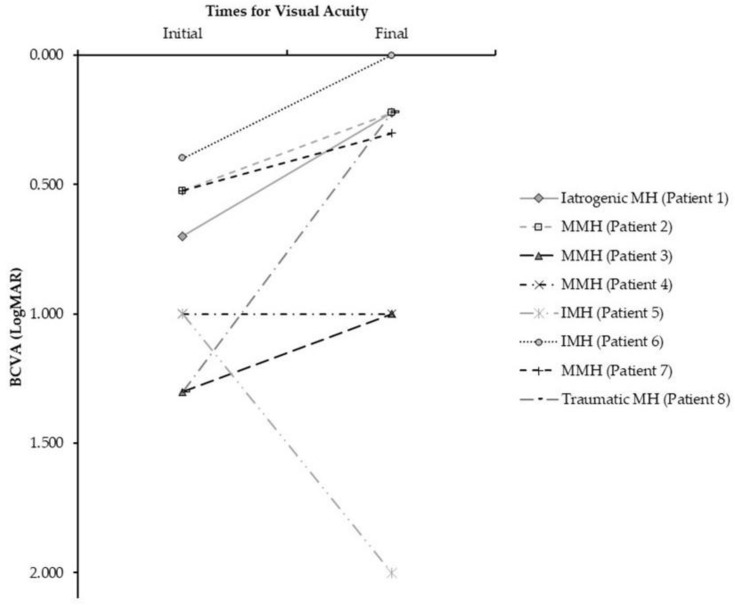
Result in visual acuity due to the use of PRGF in macular hole surgery. Description of changes in visual acuity according to the type of macular hole. PRGF: Plasma Rich in Growth Factors, BCVA: Best Corrected Visual Acuity, MMH: Myopic Macular Hole, IMH: Idiopathic Macular Hole.

The overall post-surgical follow-up time was 27.2 ± 9.01 (12.5–35.8) months, and for the MMH group it was 21.1 ± 8.84 (12.5–31.0) months. The final state of the lens was 75% (*n* = 6) pseudophakic eyes, unchanged compared to the preoperative situation. Anatomical closure of the MH was obtained in seven cases (87.5%). In one case of IMH, there was no closure of MH. However, a decrease in the anatomical measurements of the MH observed on the OCT was achieved (Table 2). Visual acuity improved in six cases (75%). The MMH group improved on average two lines of vision, one eye with closed MMH kept the BCVA unchanged, and patient number 5 had no improvement in visual acuity, and no additional surgeries were performed on this patient (Figure 1).

**Figure 2:**
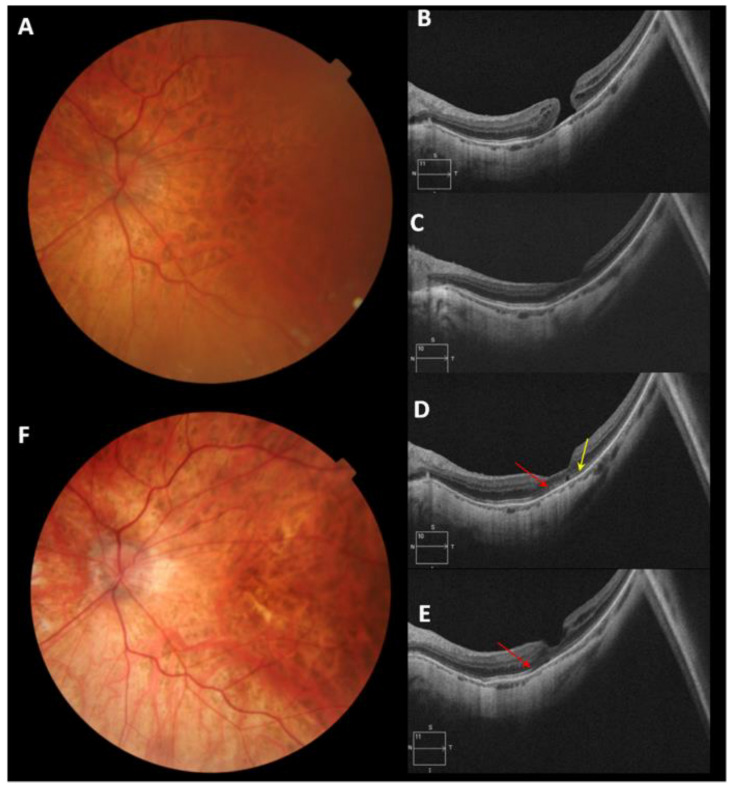
Patient number 2 diagnosed with recurrent MMH (myopic macular hole) with two previous failed PPV (Pars Plana Vitrectomy). (**A**) Retinography of recurrent MMH with 63 months of evolution, before surgery with Plasma Rich in Growth Factors liquid injection (iPRGF). (**B**)Pre-surgical optical coherence tomography (OCT) of MMH. (**C**) OCT macular image one month after surgery. (**D**) OCT at six months follow-up, restoration of the (ELM) External Limiting Membrane (red arrow) and the ellipsoid layer (yellow arrow) is observed, leaving a small cyst at the central foveal level. (**E**) OCT at twelve months of follow-up, the formation of the ELM (red arrow) is observed. (**F**) Retinography at the end of follow-up (29.5 months).

**Figure 3:**
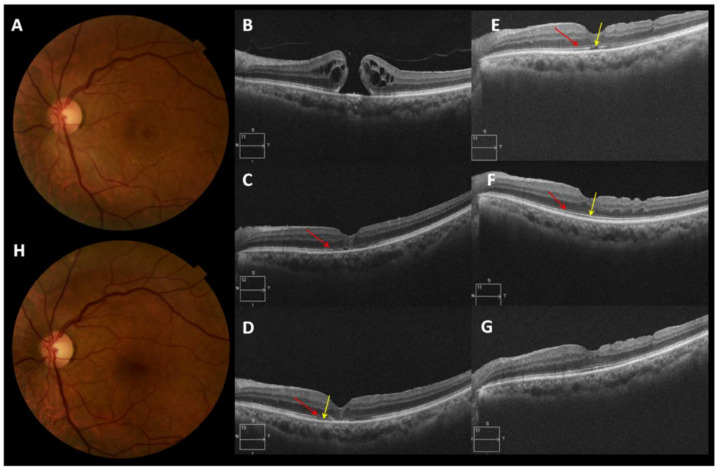
Patient number 6 diagnosed with a large idiopathic macular hole (IMH) without prior PPV (Pars Plana Vitrectomy). (**A**) Presurgical retinography of IMH in Gass stage 4 and >400 µm minimum diameter. (**B**) The initial optical coherence tomography (OCT) image shows a large IMH with multiple cystic spaces. (**C**) Image of macular OCT one month after surgery (injectable liquid and membrane PRGF were used as an adjuvant to PPV) shows the closed IMH and the start of the formation of ELM (External Limiting Membrane) (red arrow). (**D**) OCT two months post-surgery shows the formation of the ELM (red arrow) and initiates restoration of the ellipsoid layer (yellow arrow). (**E**) OCT seven months post-surgery shows the complete formation of the ELM (red arrow) at the foveal level with a lack of continuity in the ellipsoid layer (yellow arrow). (**F**) OCT 24 months post-surgery, the complete formation of the ELM (red arrow) and ellipsoid layer (yellow arrow) is observed. (**G**) OCT 35.5 months post-surgery, patient with final BCVA of 1.0 decimal (0.000 LogMar). (**H**) Retinography at the end of follow-up: no clinical evidence of IMH.

**Figure 4:**
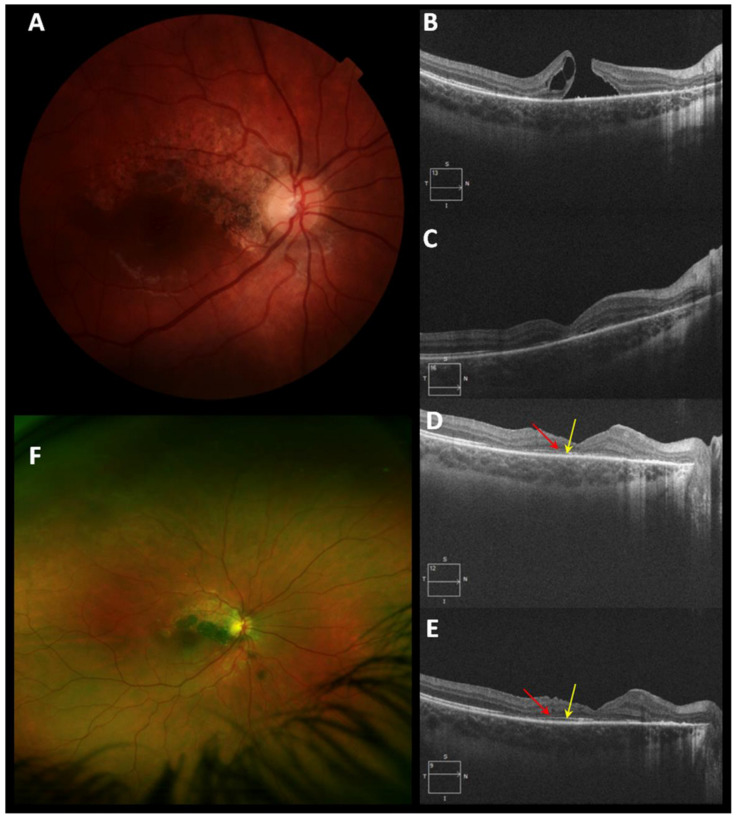
Patient number 8 with a Diagnosis of traumatic macular hole (TMH). (**A**) Retinography that shows chronic changes in the TMH of six months of evolution, no surgery has been performed. (**B**) Optical coherence tomography pre-surgical OCT. The initial BCVA (Best Corrected Visual Acuity) was 0.05 decimal (1301 LogMar), injectable liquid and PRGF membrane are used as adjuvants in PPV (Pars Plana Vitrectomy). (**C**) OCT one month after surgery. TMH closure observed. (**D**) OCT three months post-surgical. The segmented restoration of the External Limiting Membrane (ELM) (red arrow) and of the ellipsoid layer (yellow arrow) is observed. (**E**) OCT image at twelve months post-surgical follow-up, thinning of the inner layers of the retina is observed, the restoration of the ELM (red arrow) and the ellipsoid layer (yellow arrow) is maintained. (**F**) Fundus photography taken with Optomap at 35.8 months of follow-up, TMH closure is maintained, and BVCA is recovered at 0.6 decimal (0.222 LogMar).

The BCVA of the patient with iatrogenic MH improved four lines of visual acuity. Patient number 6 with IMH obtained anatomical closure, and the final BCVA was 1.0 decimal with improvement of four lines of visual acuity. In traumatic MH, the BCVA improved in six lines of visual acuity. Figure 2, Figure 3 and Figure 4 illustrate the anatomical results of patients with MH of different etiology undergoing PPV and the use of PRGF as an adjuvant. Figure 2 corresponds to patient 2 with MMH, Figure 3 illustrates the evolution of patient 6, who has an IMH, and Figure 4 represents the anatomical changes of the traumatic MH of patient number 8. During the follow-up time, no adverse events related to the given therapy were recorded.

## 4. Discussion

It is known that the anatomical closure rate of the medium-sized MH (250–400 µm), at which PPV is performed associated or not with ILM peeling, is greater than 90% [9,25]. However, for large MHs (>400 µm), the closing rates can be around 75% [27]. In many cases, this anatomical closure is not accompanied by improvement in visual acuity. 

The standard treatment for MH is PPV, posterior hyaloid removal, with/without ILM peeling, long-lasting gas tamponade, and face-down position. However, in large, myopic, chronic, or recurrent HMs, the treatment is more diverse and changes rapidly [2]. In MMH treatment, the P-ILM is controversial since this procedure may increase the risk of damage to the retinal nerve fibre layer-ganglion cell complex [28]. Different methods have been used to increase the MH closure rate, such as inverted ILM flap, inverted ILM flap insertion, multiple free ILM flap insertion, posterior lens capsule, relaxing retinotomies, including autologous neurosensory retinal flaps [29,30,31,32]. With these techniques, different clinical results are achieved. Some authors have described retinal fibrosis and dystrophy of the RPE in the macular area after autologous transplantation of ILM, which would be related to the non-recovery of visual acuity [33]. A technique described in the treatment of MMH is the macular buckling that is used in recurrent cases, with a success rate in anatomical closure between 70% and 100% according to published series [2]. However, the surgeon must be highly skilled in positioning the buckle correctly and perform the posterior suture. 

Since the 1990s, blood derivatives (autologous serum, autologous platelet concentrates (APC) or also called platelet rich plasma (PRP)) have been used classically as an adjuvant therapy to increase the rate of closure in MHs [14,34]. More recently, biological tissues have been used as scaffolding with the ability to release growth factors, including human amniotic membrane (hAM) [35], fibrin rich plasma [36], and PRGF [23,37].

In a case series, the use of autologous serum as a chorioretinal adhesive associated with PPV and subsequent hyaloid release was evaluated in 44 patients with IMH of all stages. The average visual recovery was 2.7 lines, and MH closure occurred in 67% of cases [14]. In a clinical trial comparing the use of APC versus control as adjuvant therapy to PPV in patients with chronic IMH (stages 3 and 4), IMH closure was observed in 98% vs. 82% (*p* = 0.009). However, visual acuity did not improve [24]. Differences between autologous serum and APC have been studied in an animal model of MH, where a higher proliferation rate in cells of the outer nuclear layer was evidenced in the group treated with APC [37]. In a retrospective case series, APC was used as adjuvant therapy for PPV in patients with chronic IMH (>24 months) unable to adopt the prone position after surgery. MH closure was observed in 100% during follow-up (1–6 months), and 38% of patients reported improvement in visual acuity [38]. On the other hand, in traumatic MH, it is known that there is a waiting period between three and four months in which spontaneous anatomical closure can be achieved, particularly in holes of 100 to 200 µm. However, in large traumatic MH, surgery is necessary (PPV with or without P-ILM) to achieving a closure success rate between 67% and 85% [39]. In a series of cases of four pediatric patients (10–15 years old) who had a traumatic MH stage 3 Gass (1 to 5 months of evolution), PPV associated with adjuvant treatment with APC was performed, achieving MH closure in all cases and improving between three and seven lines of vision [40]. 

In patients with recurrent IMH, the use of APC is effective in closing MH. In a retrospective study that included 61 eyes, MH closure was achieved in 85.2% of cases. However, it can be determined that patients with a defect in the ellipsoid area had worse postoperative visual acuity [41]. In another study that included 29 patients with recurrent IMH, PPV with P-ILM and gas tamponade without adjuvant therapy was performed, and MH closure was achieved in only 69% of cases [42]. 

In patients with MMH, MH closure is less effective with the standard surgical technique, the closure rate can vary between 62.5 and 87.5 according to the different published series, and the need for additional surgeries is much higher [10,11]. However, in a study that included seven eyes with MMH, using an autologous platelet-rich plasma with the open technique [11], MH closure was achieved in all cases and improved one line of visual acuity in three of the seven cases, with a mean follow-up of 5.8 months. In cases with recurrent MH in which P-ILM has already been performed and in a second PPV, it is impossible to extend the rhexis. One option is to touch the edges of the MH trying to introduce the glial tissue into the MH and to use APC as adjuvant treatment [43]. In a study with 27 MMH patients, the autologous blood clot associated with MLI replacement was evaluated to repair MH retinal detachment; anatomical closure was obtained in 96% of cases with follow-up of only 12.4 ± 5.3 months [44]. In our study, the follow-up was 27.2 ± 9.0 months, and anatomical closure was achieved in 87.5% of the cases in patients with previous vitrectomy and poor visual prognosis.

The use of the hAM as a plug in the subretinal space for the treatment of recurrent IMH has recently been published. In all eight cases evaluated, MH closure was achieved in all, improving from a mean initial BCVA of 1488 (LogMAR) to a mean final BCVA of 0.480 (LogMAR). Restoration of the outer retinal layers was also observed, with growth over the hAM plug (evaluated by OCT), which would be related to the improvement of visual acuity [35]. In this series of patients, standard tamponade gas or silicone oil was used at the end of the surgery. This suggests the need for tissue scaffolding to achieve good retinal tissue restoration.

PRGF is a select type of PRP that uses a standardized procedure called Endoret^®^ -PRGF^®^ [16,17]. Among the characteristics of the PRGF are its closed manufacturing technique, the quality marking by the European Medicines Agency (EMA), the patented platelet activation, the product versatility (injectable liquid, eye drops, clot, membrane), rapid manufacturing (in less than 60 min), the possibility of preparing it in the same surgery room, a concentration of growth factors higher than other blood derivatives, the absence of leukocytes, the inactivation of inflammatory products (complement cascade), and decreased levels of IgE [18,26]. The clinical efficacy of PRGF has been proven in ophthalmological pathologies (dry eye, neurotrophic corneal ulcers, surgical use in the conjunctiva and cornea, among others), with pre-clinical evidence supporting the proliferation, migration, and restoration of ocular tissues [19,20,21]. Particularly in MH surgery, two studies have already been published. The first was a case of recurrent MMH with 700 µm (minimum diameter) in which PPV was performed with mPRGF and a six-month follow-up, observing the closure of the MH and visual recovery of counting fingers to 0.1 (decimal) [23]. In the other study, two cases with persistent IMH with sizes of 691 µm and 1020 µm were evaluated, in which PPV was performed associated with mPRGF and iPRG. The closure of MH was observed in the two cases (12 months follow-up) and with visual acuity improvement (case 1:1 to 0.69 (LogMAR), case 2:1.8 to 0.64 (LogMar)) [45].

Our study evaluated the anatomical and functional outcome of mPRGF and iPRGF as an adjuvant to PPV in patients with recurrent, persistent or with poor prognosis MH of various etiologies. Anatomical closure was observed in seven cases (87.5%), and visual acuity improvement in six cases, particularly in the MMH group, which improved two lines of vision. The most frequent cases were MMH (*n* = 4.50%), followed by IMH (*n* = 2.25%), one case of idiopathic MH, and one traumatic. The MMH patients were also chronic since they had an average of more than 24 months until the new surgery with PRGF. In one case, MH closure was not obtained, perhaps due to its large size (1577 µm). If the first surgery had involved the use of mPRGF plus iPRGF as adjuvant therapy, it is possible that closure of the macular hole would have been achieved. No adverse events were recorded with PRGF, and the overall follow-up time was long (27.2 months on average), which shows clinical stability and no reopening of HM. The use of mPRGF as scaffolding for the restoration of retinal tissue, associated with iPRGF with the gradual release of growth factors, can be a great therapeutic option since the regeneration of the outer layers is evidenced in this study.

The PRGF contains among its molecules PDGF (platelet-derived growth factor), TGF- β (transforming growth factor-β), FGF (fibroblast growth factor), EGF (epidermal growth factor), IGF (insulin-like growth factor), NGF (nerve growth factor), and other growth factors, which are involved in cell proliferation, modulation of inflammation and tissue restoration [16,17]. On the other hand, the mPRGF provides a sustained release of growth factors and has mechanical properties that allow its use in surgery [21,46,47]. At the same time, the efficacy of the use of mPRGF as scaffolding has been seen in research models of advanced therapies in cell expansion [48]. The mechanisms involved in the closure of MH when using PRGF include trophic action of growth factors [23,49] as well as Müller cell migration and concentration [11]. Moreover, mPRGF allows the sustained release of growth factors, promotes glial growth (not fibrosis) [35,45,50], and modulates the antioxidant response in the RPE [51].

There are some disadvantages in the manufacturing of ACP or PRPs, such as open production, which requires the use of a laminar flow hood, the availability of a hematology service for the elaboration of the products, and some of these products may most likely contain leukocytes, which increases the inflammatory response. Moreover, such use as part of a non-standardized manufacturing technique limits clinical predictability [17]. In the case of the hAM, it requires the availability of a tissue bank and highly specialized personnel for production, which increases the associated costs. 

In this study, the use of PRGF as adjuvant therapy for vitrectomy in MHs of different etiologies (IMH, MMH, iatrogenic and traumatic) with poor prognosis, persistent or recurrent, has been evaluated. This autologous biological approach may be a new tool that can be added to standard surgical techniques in macular surgery. Our study has some limitations, such as being retrospective, not comparative, of different MH etiologies, and with a small sample. However, it opens the door to the design of future clinical trials in which the findings reported in this publication can be more strongly confirmed. Our study reinforces the hypothesis of the concept of PRGF-mediated retinal regeneration, in which pre-clinical and clinical research should be further expanded. 

## 5. Conclusions

In the patients of this pilot study, efficacy was observed in closing the MH and improving visual acuity. This publication could open the door to the use of PRGF as an adjuvant therapy in the surgery of recurrent, persistent, or poor prognosis MH. These findings need to be confirmed with further comparative studies.

## Figures and Tables

**Table 1:** Baseline characteristics of patients with macular hole.

Num. Patient/Age (Years)/Gender	Laterality (Eye)	Primary Ophthalmologic Disease	MH Etiology	Time with Diagnosis of MH (Months)	Number of Previous Surgeries	Detail of Previous Surgeries	MH Time Open Since Last Surgery (Months)
1/36/M	R	PDR + VH + SMH	Iatrogenic	3	1	1st: PPV + P-ILM + C3F8	1.6
2/64/F	L	High myopia	MMH	72	2	1st: PPV + TMHE + C3F8; 2nd: PPV + TMHE + C3F8	63
3/47/M	R	High myopia	MMH	3	1	1st: PPV + P-ILM + C3F8	2.8
4/62/M	L	High myopia	MMH	24	1	1st: PPV + P-ILM + C3F8	21.5
5/71/M	R	Primary MH	IMH	3	1	1st: PPV + P-ILM + C3F8	2
6/68/F	L	Primary MH	IMH	24	0	N/A	24
7/62/F	L	High myopia	MMH	24	2	1st: PPV + TMHE + C3F8; 2nd: PPV + TMHE + C3F8	12
8/15/M	R	Traumatic MH	Trauma	6	0	N/A	6

M: Male, F: Female, R: Right, L: Left, MH: Macular Hole, MMH: Myopic Macular Hole, IMH: Idiopathic Macular Hole, PDR: Proliferative Diabetic Retinopathy, VH: Vitreous Hemorrhage, PPV: Pars Plana Vitrectomy, SMH: Subhyaloid Macular Hemorrhage, TMHE: Touch of Macular Hole Edges, P-ILM: Peeling of Internal Limiting Membrane, N/A: Not applicable.

**Table 2:** Pre-surgical data and post-surgery results.

Patient	Pre-Surgery Lens	BCVA Pre-Surgical; Decimal (LogMAR)	IOP Pre-surgical (mmHg)	Base Diameter of MH (µm)	Minimum Diameter of MH (µm)	Height of MH (µm)	Surgery Performed	Final State of the Lens	BCVA Final; Decimal (LogMAR)	IOP Final (mmHg)	Follow-Up Time (Month)	Final Closure of MH
1	Phakic	0.2 (0.699)	14	1031	433	514	PPV + rexis ILM + TMHE + iPRGF + C3F8	Phakic	0.6 (0.222)	14	29	Si
2	Pseudophakic	0.3 (0.523)	15	673	313	389	PPV + TMHE + iPRGF + C3F8	Pseudophakic	0.6 (0.222)	11	25.9	Si
3	Pseudophakic	0.05 (1.301)	12	1345	806	497	PPV + R and T ILM + m/iPRGF + SilOil	Pseudophakic	0.1 (1.000)	15	12.5	Si
4	Pseudophakic	0.1 (1.000)	13	1470	633	511	PPV + rexis ILM + TMHE + m/iPRGF + C3F8	Pseudophakic	0.1 (1.000)	12	14.8	Si
5	Pseudophakic	0.1 (1.000)	13	1577	499	473	PPV + R and T ILM + m/iPRGF + C3F8	Pseudophakic	0.01 (2.000)	9	33.3	No †
6	Pseudophakic	0.4 (0.398)	15	1209	547	519	PPV + P-ILM + m/iPRGF + C3F8	Pseudophakic	1.0 (0.000)	15	35.5	Si
7	Pseudophakic	0.3 (0.523)	13	710	374	419	PPV + TMHE + iPRGF + C3F8	Pseudophakic	0.5 (0.301)	12	31	Si
8	Phakic	0.05 (1.301)	17	1438	530	380	PPV + P-ILM + m/iPRGF + C3F8	Phakic	0.6 (0.222)	13	35.8	Si

BCVA: Best Corrected Visual Acuity, IOP: intraocular pressure, MH: Macular Hole, PPV: Pars Plana Vitrectomy, ILM: Internal Limiting Membrane, TMHE: Touch of Macular Hole Edges, iPRGF: Plasma Rich in Growth Factors in injectable liquid form, P-ILM: Peeling of Internal Limiting Membrane, R and T ILM: Rexis and transposition of internal limiting membrane inside the macular hole, m/iPRGF: Plasma Rich in Growth Factors in Membrane form (100 µm) placed inside the macular hole and associated with injectable liquid PRGF, SilOil: Silicone Oil, ERM: Epiretinal membrane, † Macular hole not closed (remains with opening base diameter: 960 µm, minimum diameter: 490 µm and height: 364 µm).

## Data Availability

All data were fully anonymized and are available upon request.
